# COMMENTARY ON: “MAPPING THE COMMONALITIES OF REHABILITATION: USING GUIDE-REHAB TO OPEN THE BLACK BOX OF PROGRAMMES ACROSS DIVERSE HEALTH CONDITIONS”

**DOI:** 10.2340/jrm.v58.46449

**Published:** 2026-08-17

**Authors:** Paolo BOLDRINI, Mauro ZAMPOLINI

**Affiliations:** 1 Physical and Rehabilitation Medicine Specialist, Private Practice, Ferrara, Italy; Member of the Executive Committee, European Society of Physical and Rehabilitation Medicine (ESPRM); 2 Director, Department of Rehabilitation Medicine, USL UMBRIA 2, Italy; President of UEMS PRM Section

Rehabilitation has long suffered from a peculiar paradox. It is, by international consensus, a single, cross-cutting health strategy – one of the fundamental pillars of health systems (1). Despite this statement, the common characteristics that should define rehabilitation as a specific entity applicable to various clinical conditions have not yet been clearly identified. Rehabilitation seems fragmented into as many “rehabilitations” as there are diagnoses, settings, and professional traditions. The paper by Negrini and colleagues, “Mapping the commonalities of rehabilitation: using GUIDE-Rehab to open the black box of programmes across diverse health conditions”, (2) is a conceptually important attempt to close that gap.

The study takes 7 rehabilitation programmes from the PREPARE consortium (3) and asks a question rarely posed explicitly: beyond their obvious clinical differences, do these programmes share an underlying architecture? The authors do not compare diagnoses or outcomes but provide *descriptions*, using an adapted version of GUIDE-Rehab (4) to decompose each programme into targets, components, active ingredients, and intervention theories, and then map these onto the domains of the International Classification of Functioning, Disability and Health (ICF).

This is the paper’s principal contribution. Rehabilitation has been characterized as a “black box” (4): multimodal, person-oriented, collaborative, and resistant to the itemized reporting that drug trials allow almost by default. Neither this multidimensionality nor the difficulty of building a taxonomy to open the black box is new. For example, decades ago Whyte (5) argued that rehabilitation needed an explicit research methodology; that line was later formalized in the “Rehabilitation Treatment Specification System” (6), which holds that treatments cannot be classified by surface appearance and require explicit specification of ingredients and targets to be compared across studies. Other authors pursued a convergent line, first showing empirically that clinical replicability of rehabilitation interventions in major journals is inadequate, then demonstrating, in a review of “usual care” in stroke control groups, that the term conceals not a black box but a “black hole” – a comparator too poorly specified to characterize at all (7). Read against this trajectory, the present paper is less a new claim than a demonstration of what a shared taxonomy can finally do: turn decades of argument over indescribable interventions into a concrete, comparable picture. A related, underappreciated contribution concerns the ICF itself: on its own, it does not ensure that 2 raters classify the same component in the same way, and its domains have often been applied impressionistically. By forcing components, ingredients, and targets through GUIDE-Rehab’s decomposition before ICF coding, the study shows how the ICF can be operationalized consistently across raters, conditions, and settings – which arguably allows 7 unrelated programmes to be placed, for the first time, on the same analytic map. An interesting point is that, by decomposing programmes into targets, components, and ingredients rather than by professional label, GUIDE-Rehab quietly erodes the “non-pharmacological, non-surgical” boundary by which rehabilitation has often defined itself. This is not a flaw in the method but one of its more intriguing contributions: it suggests that the unit of analysis in rehabilitation should be the function-oriented goal and its active ingredients, not the discipline that happens to deliver them – a reframing with direct consequences for how services are organized, reimbursed, and counted.

The results of this study may reassure, and simultaneously unsettle, clinicians working across specialities. They reassure because they confirm empirically that programmes for entirely unrelated conditions draw on a shared repertoire. They unsettle because they expose a systemic imbalance: collectively, the 7 programmes touch every ICF domain, but individually, none does. Body Functions and Structures dominate; Participation – arguably the domain that matters most to patients – is explicitly addressed by only 1 partner, and Contextual Factors fare little better. Clinicians have long suspected rehabilitation drifts toward impairment-level targets under pressure of measurable, short-term outcomes; GUIDE-Rehab now lets this drift be demonstrated and located precisely, showing it concentrated unevenly programme by programme rather than being absent from the field.

This gap runs directly counter to rehabilitation’s stated purpose of promoting participation, and several non-exclusive explanations seem plausible. The most obvious is the residual dominance of the biomedical model, which still treats body-level impairment as the primary, measurable, reimbursable target. At the same time, participation is left to be addressed informally or assumed to follow once function improves. A second is organizational: most of the 7 programmes are delivered in discipline-specific settings – orthopaedic, surgical, or outpatient clinics – that are structurally ill-suited to addressing environmental and social barriers that lie outside the clinic walls and require intersectoral coordination that few services are resourced to provide. A third, more conceptual explanation is that Participation and Contextual Factors remain difficult to operationalize as treatment targets: unlike strength or range of motion, they lack agreed, intervention-linkable measures, so clinicians and trialists default to what can be specified and quantified. These explanations are not mutually exclusive and disentangling them – itself an interesting use case for GUIDE-Rehab applied prospectively – would clarify whether the imbalance is a problem of will, of organization, or of measurement science.

A methodological caveat deserves attention. The 7 programmes differ along 3 axes at once – health condition, care setting, and organizational/health-system context – and because each partner contributes 1 condition embedded in 1 setting embedded in 1 health system, these axes are fully confounded. A skew toward Body Functions in 1 programme could reflect the condition itself, the setting, or local staffing and reimbursement arrangements, and the design cannot discriminate between these. Future research should hold the condition constant while varying the setting and context – for example, by comparing stroke rehabilitation profiles across inpatient, outpatient, and community services, and across differently organized health systems.

The authors are appropriately candid about limitations: a small, self-selected, heterogeneous sample; a restricted subset of GUIDE-Rehab items; reliance on 1 rater’s categorization, checked by a second; and pilot feedback confirming the tool, however logical, is “difficult and time-consuming” to apply. These do not undermine the achievement – tools that make complexity visible are seldom effortless to use. The more interesting open question is not whether GUIDE-Rehab works, but whether the heterogeneity it has now made visible reflects genuine clinical necessity or an artefact of condition-specific reporting habits that a common framework could correct. Answering that, through prospective, multi-condition application of the full GUIDE-Rehab, would move rehabilitation closer to the comparable, function-oriented evidence base other complex-intervention fields have begun to build.

This connects to a practical concern the authors themselves raise in routine clinical work: completing GUIDE-Rehab item by item is burdensome, and this burden – not any conceptual flaw – is the likeliest barrier to adoption. Artificial intelligence, particularly natural language processing, is unlikely to stay peripheral here. Beyond automating extraction of ingredients and targets from clinical records and linking them to ICF codes, as the authors propose, such tools could eventually scan many programme descriptions across conditions and surface recurring components and target patterns that no manual 7-programme comparison could detect at scale – supporting, rather than merely lightening, the search for cross-condition commonalities this study has so far pursued by hand.

This growing dependence on AI tools, however, cuts both ways: it is also a reminder that clinicians will increasingly need working literacy in how these systems extract, classify, and aggregate clinical information, not merely as researchers but in routine practice. Understanding what an NLP pipeline can and cannot reliably infer from a clinical note – and where it might silently misclassify a target or an ingredient – will matter as much as understanding GUIDE-Rehab itself, and rehabilitation training programmes should begin building this competence now rather than treating it as a specialist concern for the future.

*The authors used an AI-assisted writing tool (Claude, Anthropic) to support the drafting and stylistic revision of this commentary. All content, arguments, and interpretations were conceived, directed, and critically reviewed by the authors, who take full responsibility for the accuracy and integrity of the final text.*

## References

[1] World Health Assembly. Strengthening rehabilitation in health systems. Resolution WHA76.6. Geneva: World Health Organization; 2023 [accessed Jun 26, 2026] Available from: https://apps.who.int/gb/ebwha/pdf_files/EB152/B152%2810%29-en.pdf

[2] Negrini S, Burger H, Capodaglio P, Ceravolo MG, Hoogendam L, Janssen E, et al. Mapping the commonalities of rehabilitation: using GUIDE-Rehab to open the black box of programmes across diverse health conditions. J Rehabil Med 2026; 58: jrm46211. 10.2340/jrm.v58.46211 42605920 PMC13488149

[3] PREPARE Consortium. Personalised REhabilitation via Novel AI PAtient StRatification StratEgies (PREPARE); 2023 [accessed Jun 26, 2026]. Available from: https://cordis.europa.eu/project/id/101080288 10.3233/SHTI260732 42290409

[4] Negrini S, Arienti C, Armijo-Olivo S, Côté P, Heinemann AW, Kiekens C, et al. Reporting GUideline for Intervention DEscription in Rehabilitation (GUIDE-Rehab): a tool to open the ‘black box’ of rehabilitation complex interventions. BMJ Evid Based Med 2025: bmjebm-2025-113997. [Online ahead of print] 10.1136/bmjebm-2025-113997 41390170

[5] Whyte J. Toward a methodology for rehabilitation research. Am J Phys Med Rehabil 1994; 73: 428–435. 10.1097/00002060-199411000-00008 7993617

[6] Van Stan JH, Dijkers MP, Whyte J, Hart T, Turkstra LS, Zanca JM, et al. The Rehabilitation Treatment Specification System: implications for improvements in research design, reporting, replication, and synthesis. Arch Phys Med Rehabil 2019; 100: 146–155. 10.1016/j.apmr.2018.09.112 30267666 PMC6452635

[7] Arienti C, Buraschi R, Pollet J, Lazzarini SG, Cordani C, Negrini S, et al. A systematic review opens the black box of “usual care” in stroke rehabilitation control groups and finds a black hole. Eur J Phys Rehabil Med 2022; 58: 520–529. 10.23736/S1973-9087.22.07413-5 35634889 PMC9980563

